# New Insights into Mechanisms of Cisplatin Resistance: From Tumor Cell to Microenvironment

**DOI:** 10.3390/ijms20174136

**Published:** 2019-08-24

**Authors:** Shang-Hung Chen, Jang-Yang Chang

**Affiliations:** 1 National Institute of Cancer Research, National Health Research Institutes, Tainan 70456, Taiwan; 2 Division of Hematology/Oncology, Department of Internal Medicine, National Cheng Kung University Hospital, College of Medicine, National Cheng Kung University, Tainan 70101, Taiwan

**Keywords:** cisplatin, tumor microenvironment, drug resistance

## Abstract

Although cisplatin has been a pivotal chemotherapy drug in treating patients with various types of cancer for decades, drug resistance has been a major clinical impediment. In general, cisplatin exerts cytotoxic effects in tumor cells mainly through the generation of DNA-platinum adducts and subsequent DNA damage response. Accordingly, considerable effort has been devoted to clarify the resistance mechanisms inside tumor cells, such as decreased drug accumulation, enhanced detoxification activity, promotion of DNA repair capacity, and inactivated cell death signaling. However, recent advances in high-throughput techniques, cell culture platforms, animal models, and analytic methods have also demonstrated that the tumor microenvironment plays a key role in the development of cisplatin resistance. Recent clinical successes in combination treatments with cisplatin and novel agents targeting components in the tumor microenvironment, such as angiogenesis and immune cells, have also supported the therapeutic value of these components in cisplatin resistance. In this review, we summarize resistance mechanisms with respect to a single tumor cell and crucial components in the tumor microenvironment, particularly focusing on favorable results from clinical studies. By compiling emerging evidence from preclinical and clinical studies, this review may provide insights into the development of a novel approach to overcome cisplatin resistance.

## 1. Introduction

Cisplatin (CDDP), a platinum-based anticancer agent, is one of the most commonly used chemotherapy drugs in the treatment of patients with various types of cancer, such as bladder, ovarian, head and neck, lung, testicular, cervical, esophageal, and breast cancer [1]. CDDP and its analogs, carboplatin and oxaliplatin, have been shown to have significant efficacy in cancer treatment with both curative and palliative intent [2]. In addition to monotherapy, the anticancer activity of CDDP has also been demonstrated when it is used in combination with other types of agents in chemotherapy, radiotherapy, and even immunotherapy [3]. Therefore, CDDP continues to play an active role in systemic anticancer treatments in this modern era full of drugs with specific targets and immunogenic therapies. To date, it is estimated that approximately 50% of all patients with cancer will be treated with CDDP in their anticancer therapies [3]. Determining how to improve the clinical utility of CDDP and other platinum-based drugs has attracted close attention from worldwide researchers in oncology, pharmacology, and chemistry.

The molecular structure of CDDP contains the basic platinum complex, two amides, and two chloride ligands in a cis elemental orientation. This first metal-based chemotherapy drug was discovered by the Italian chemist Michele Peyrone in 1845, and its ability to inhibit cell division was recognized by the American biophysicist Dr. Barnett Rosenberg in 1965. Through continuous efforts devoted to working on preclinical and clinical studies, the U.S. Food and Drug Administration approved CDDP use for the treatment of patients with testicular and bladder cancer in 1978 [3]. Since then, CDDP treatment has exhibited a high degree of activity against tumor growth in various solid cancers, and millions of patients have received this chemotherapy drug in their anticancer therapies. Despite major clinical success, drug resistance of tumor cells has hindered the clinical utility of CDDP for decades [4]. The binding of platinum to DNA is the major cytotoxic mechanism after CDDP entry into tumor cells. Accordingly, conventional studies of CDDP resistance have largely focused on intracellular functions in mediating drug accumulation, DNA damage repair, and apoptotic signaling pathways. However, the growth of tumor cells is a dynamic biological process, which entails close interaction with the surrounding environment, especially when these cells are confronted with external stress from chemotherapy drugs [5]. Increasing evidence has demonstrated that the tumor microenvironment (TME) is also important in the development of resistance to chemotherapy drugs, thereby affecting their therapeutic effectiveness in tumor cells [6,7]. In this review, we present brief and to-the-point summaries concerning CDDP resistance in tumor cells and the TME. These discussions focus on experimental evidence with clinical implications.

## 2. Mechanism of CDDP-Induced Cytotoxicity

In addition to the passive diffusion mechanism, platinum-based chemotherapy drugs can enter or exit cells through the transmembrane transportation system [8]. After entering cells, CDDP activity is first initiated by the reaction of aquation [9]. Owing to the relatively low concentration of chloride ions in the cytosol, CDDP aquation occurs spontaneously and leads to the substitution of one or both chloride ligands with water molecules. These aquated forms of CDDP are highly reactive with numerous cytoplasmic substrates, such as reduced glutathione (GSH) and metallothionein (MT) proteins [10,11]. Because of a nucleophilic propensity, nuclear DNA is also the main target prone to react with aquated CDDP [12]. Additionally, the N7 position of the guanine base is the preferred site of attack of aquated CDDP rather than other bases present in DNA. Several types of DNA adducts can be generated from the interaction between CDDP and DNA bases, such as monoadducts, intrastand crosslinks, and interstand crosslinks. If only a small number of DNA damage lesions are formed, all these platinum-DNA adducts can be totally eliminated by the intimate cooperation of DNA repair systems in cells [13]. By contrast, when the extent of CDDP-induced DNA damage exceeds repair capacity, cells will proceed to their deaths, most often through the activation of the apoptotic signaling pathway [14,15]. These cytotoxic mechanisms of CDDP based on cellular biology underpin the research map of tumor resistance to this platinum-based drug.

## 3. Conventional Perspectives on CDDP Resistance from a Tumor Cell

**Figure 1:**
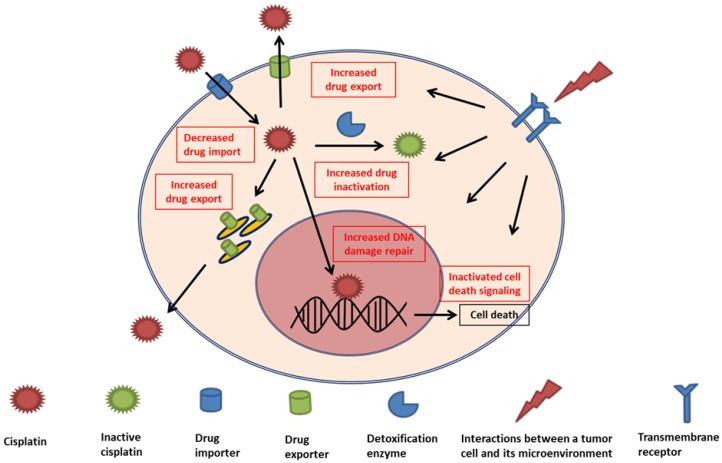
Schematic representation of intracellular mechanisms with an effect on the development of cisplatin resistance with clinical implications. Inside the tumor cell, decreased drug import, increased drug export, increased drug inactivation by detoxification enzymes, increased DNA damage repair, and inactivated cell death signaling are major mechanisms leading to cisplatin resistance. Interactions between a cell and its environmental components can also promote these internal mechanisms and subsequent cisplatin resistance.

Because the principal action mechanism of CDDP cytotoxicity is the formation of platinum-DNA adducts, cellular reactions that attenuate this type of DNA damage are key factors in the development of resistance to this metal drug. From the most accepted perspectives, three intracellular adaptation mechanisms have been proposed to be primarily responsible for the development of CDDP resistance [1,3,4]. These responsible mechanisms, summarized in Figure 1 and Table 1, are alterations in cellular accumulation of the drug, intracellular detoxification of the drug, and DNA damage repair. Although relative differences of each mechanism contribute to overall tumor resistance, more than one mechanism can be present simultaneously in tumor cells resistant to platinum-based drugs [4].

### 3.1. Cellular Accumulation of Drug

Tumor cells attenuate the formation of platinum-DNA adducts through reduced cellular accumulation of the drug. Several in vitro and clinical studies have demonstrated that platinum concentrations in tumor cells or tissues are correlated with CDDP resistance [4,16,17,49,50]. The reduction in cellular CDDP accumulation can be the functional result of two independent pathways regulating intracellular drug uptake and export. We present some perspectives on these mechanisms as follows.

#### 3.1.1. Decrease in Uptake

Passive diffusion and transmembrane transportation systems control the influx of CDDP into a cell. Previous studies have indicated that copper (Cu) transporters principally participate in the import and export of platinum-based agents [4,16,17,50]. Among these transporters, Cu transporter 1 (CTR1), a highly conserved Cu importer, plays a potent role in cellular uptake of CDDP [4,50]. In vitro and clinical studies have determined that CTR1 overexpression can increase CDDP uptake in lung cancer cells [18,19]. Our previous studies have also revealed that low expression levels of CTR1 are observed in cervical cancer cells resistant to platinum-based drugs [16,17]. However, cotreatment with a Cu chelator, D-penicillamine, and CDDP can result in increased expression levels of CTR1 and a synergistic cytotoxic effect in our CDDP-resistant cervical cancer cells [16]. In our recent study, we also found that an iron-chelating agent, desferal, can reduce CDDP-resistance through the regulation of CTR1 and transferrin receptor 1 [17]. In a xenograft study, Ishida and colleagues reported that a Cu chelator, tetrathiomolybdate, can enhance CDDP efficacy dependent on the function of CTR1 in cervical cancer [20]. In a research analyzing clinical tumor tissues, Yang and colleagues also demonstrated that high expression levels of CTR1 correlate with favorable prognoses in patients with lung cancer receiving platinum-based therapies [21]. All these results support the vital role of this Cu transporter in cellular resistance to CDDP.

Some ion importers were reported to be correlated with CDDP resistance. The Cu transporter 2 (CTR2) is in the same family as CTR1 and primarily localized in intracellular vesicles. Recent studies have indicated that CTR2 can induce cleavage of CTR1, leading to a substantially reduced influx of CDDP [22]. In studies analyzing clinical specimens, patients with high expression levels of CTR2 in ovarian tumors had poor survival outcomes from CDDP treatment compared with those with low expression levels [23,24]. In addition, the organic cation transporter 2 (OCT2), frequently expressed in the kidney, can control cellular transportation of CDDP [51]. Naka and colleagues demonstrated that patients with gastric cancer and high expression levels of OCT2 are significantly correlated with positive responders to CDDP-based therapy [25]. However, studies on the involvement of CTR2 and OCT2 in CDDP resistance are limited in certain types of cancer, and further investigations are warranted.

#### 3.1.2. Increase in Efflux

Several studies have reported that some cellular exporters are involved in CDDP resistance, and the most notable ones are copper-transporting ATPase 1 and 2 (ATP7A and B). The principal function of ATP7A and ATP7B, which belong to the transporter family of P-type ATPases, is removing excessive Cu from cells. These ATPases located at the trans-Golgi network can also regulate the cellular efflux of CDDP [50]. In our previous studies, we have also shown that the expression levels of ATP7A are significantly higher in cervical cancer cells resistant to platinum drugs than in parenteral cells [16]. Some studies have evaluated the expression levels of these two ATPases in clinical tumor tissues and their correlations with CDDP efficacy. Studies on patients with lung and ovarian cancer have indicated that high expression levels of ATP7A are correlated with poor response to CDDP treatments and that of ATP7B have similar outcomes in patients with lung, oral, esophagus, and ovarian cancer [21,26,27,28,29].

Some studies have also suggested that multidrug resistance-associated proteins (MRPs) and members of ATP-binding cassette (ABC) transporters can mediate cellular resistance to CDDP [4]. The function of MRPs in mediating CDDP resistance is mainly achieved through the removal of platinum-GSH conjugates in an ATP-dependent manner. Among these MRPs, MRP2 is the most accepted member associated with CDDP resistance. Some studies analyzing clinical specimens have reported that high expression levels of MRP2 correlate to poor CDDP response in patients with colorectal, hepatocellular, and esophageal cancer [30,31,32]. 

### 3.2. Intracellular Drug Detoxification

As mentioned earlier, aquated CDDP has a high affinity for cytoplasmic nucleophilic species, such as GSH, MTs, and other cysteine-rich proteins [3,4]. However, CDDP activity is also limited in tumor cells after the formation of platinum-GSH conjugates. These conjugates can facilitate the excretion of CDDP through the cooperation of MRPs and therefore affect drug resistance. Furthermore, MTs in cytosol can operate in cellular metal homeostasis and detoxification upon exposure to heavy metal. Studies have indicated that CDDP-resistant tumor cells can present high expression levels of these cellular scavenger and detoxification enzymes [34,36]. Some studies examining clinical tissues have also revealed that high expression levels of glutathione S-transferase (the enzyme that assists CDDP with GSH conjugation) and MT are associated with reduced CDDP efficacy in patients with lung, ovarian, and esophageal cancer [35,37]. Furthermore, the transcription factor nuclear factor erythroid 2-related factor 2 (Nrf2) can mediate transcriptional levels of several genes that control cellular redox homeostasis, and combat harmful effects of CDDP (e.g., GSH and MRP) [52,53,54]. Previous cell-based studies have shown that tumor cells with elevated expression levels of Nrf2 are resistant to CDDP cytotoxicity. Some clinical studies analyzing tumor specimens have reported that high expression levels of Nrf2 correlate to inferior benefit from CDDP-containing therapies in patients with bladder and lung cancer [55,56]. These studies also emphasize the importance of intracellular drug efflux transporters and detoxification enzymes in mediating CDDP resistance.

### 3.3. DNA Damage Repair

To maintain the integrity of genetic substances, an intricate network is supported by each exquisite DNA repair system. Genomic DNA is the primary biological target of CDDP, and hence, cells require this network made up of DNA repair systems to remove platinum-DNA adducts. A large number of experimental studies have determined that enhanced DNA repair activity can limit tumor response to CDDP. We briefly review these critical mechanisms correlating with CDDP resistance in the following.

#### 3.3.1. Nucleotide Excision Repair

After binding to DNA, the majority of CDDP-induced DNA damage involves the formation of intrastrand platinum-DNA adducts [1,3,4,13]. The nucleotide excision repair (NER) pathway is the most recognized DNA repair system responsible for the removal of these bulky adducts. In the repair process, at least 30 proteins participate in the recognition, verification, unwinding, and excision of DNA adducts and the filling of DNA gaps after the removal of CDDP lesions. Among these NER-associated proteins, DNA excision repair protein ERCC-1 (ERCC1) is one of the most studied components involved in CDDP resistance. During the repair of CDDP-induced damage, the excision of platinum adducts executed by the ERCC1- DNA repair endonuclease XPF (XPF) dimer is the rate-limiting step in the whole NER pathway. Experimental evidence suggested that CDDP-resistant tumor cells correlated with high expression levels of ERCC1. [13]. Many studies have assessed the predictive value of ERCC1 in patients with cancer receiving CDDP-based therapies. Various reports have shown the inverse correlation between high ERCC1 expression levels and clinical CDDP efficacy in patients with ovarian [38], lung [39,40], nasopharyngeal [41], esophageal [42], cervical [43], head and neck [44], and biliary tract cancer [45]. Additionally, because the ERCC1-XPF dimer excises the platinum adducts, it is rational to examine whether XPF expression levels correlate with tumor cells resistant to platinum drugs. A study analyzing clinical specimens revealed that high expression levels of XPF are associated with inferior clinical response to CDDP in patients with head and neck cancer [46]. All these findings indicate the crucial role of NER in the repair of platinum-induced DNA damage in tumor cells.

#### 3.3.2. BRCA1/BRCA2

CDDP-induced DNA adducts without sufficient repair by the NER system can generate double-strand breaks, the most lethal DNA damage lesion. The homologous recombination (HR) system is the main cellular machinery responsible for the repair of this dangerous DNA damage type [57]. Breast cancer type 1 and 2 susceptibility protein (BRCA1 and 2) are two vital components of this system, and genetic alterations of their encoded genes are commonly found in patients with hereditary breast and ovarian cancer syndromes. Accordingly, many studies have explored the significance of these two DNA repair proteins in the clinical efficacy of DNA-damaging agents, including CDDP. For example, a study enrolling patients with *BRCA1/2*-defective ovarian cancer demonstrated that patients with these mutations respond positively to platinum-based drug therapies [47,57]. In particular, the National Comprehensive Cancer Network Guideline states that CDDP-based regimens are preferential in the treatment of patients with pancreatic cancer and known *BRCA1/2* mutations [58]. Although there were only a few enrolled subjects, a study from an eminent cancer research center demonstrated that patients with pancreatic cancer carrying these mutations had favorable responses to platinum-based treatments [48].

#### 3.3.3. Other DNA Repair Participants

In addition to the NER and HR systems, numerous studies have clarified the correlation between the mismatch repair (MMR) pathway and CDDP resistance [13]. The MMR pathway, which normally participates in the correction of single-strand DNA errors during replications, is also able to recognize the formation of platinum-DNA adducts. Thus, the MMR defect is now thought to engender the development of DNA damage tolerance and subsequent CDDP resistance. Additionally, recent studies have revealed a notable correlation between O6-methylguanine DNA methyltransferase (MGMT) expression levels and CDDP resistance [49,59]. This unique DNA repair enzyme that repairs alkyl adducts at the O6-position of guanine is a well-known regulator of cellular resistance to O6-alkylguanine alkylating agents [60]. Our previous work showed that MGMT-proficient nasopharyngeal carcinoma cells are more resistant to CDDP cytotoxicity than MGMT-deficient cells [49]. Furthermore, we demonstrated that the MGMT protein can directly bind to platinum–DNA adducts and reinforce cellular repair capacity to CDDP-induced DNA damages. After analyzing clinical outcomes of 83 patients with nasopharyngeal carcinoma receiving CDDP-based therapies, we also observed that high expression levels of MGMT in tumor specimens predict worse survival outcomes. A recent study also suggested that a high MGMT expression level can be a poor prognostic factor in patients with bladder cancer receiving platinum-based drugs as an adjuvant therapy [59]. With the clinical development of MGMT inhibitors, incorporating these inhibitors into CDDP-based therapies seems to be a novel therapeutic strategy with potential in combating drug resistance.

## 4. Emerging Perspectives on CDDP Resistance from the TME

**Figure 2:**
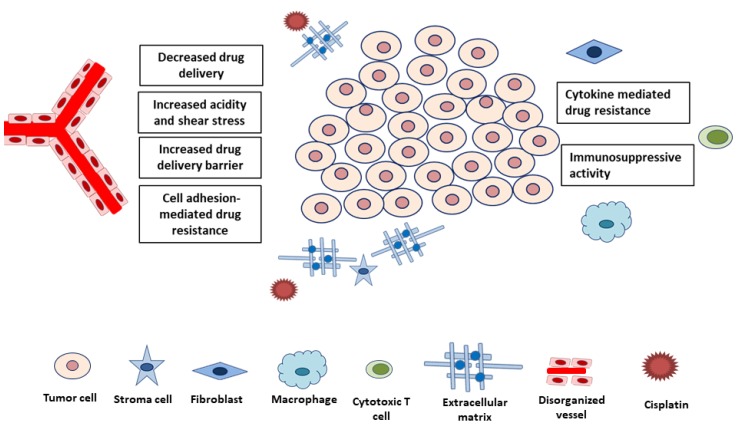
Components of tumor microenvironment reported to affect cisplatin resistance. These components can contribute to cisplatin resistance through decreased drug delivery, increased acidity or shear stress, cell adhesion or cytokine-mediated drug resistance mechanisms, and immunosuppressive activity.

Although adaptive mechanisms within cancer cells are major research topics, more recent experiments have highlighted the effects of TME on CDDP resistance [6,7]. In contrast to traditional belief of immunosuppressive effect induced by chemotherapy drugs, immunomodulatory effects regulated by CDDP are also important mechanisms of tumor cytotoxicity [61]. Emerging preclinical studies suggested that CDDP can modulate anti-cancer activity through the regulations of major histocompatibility complex class I, cytotoxic effectors, and immunosuppressive cells in the TME. Generally, the TME factors affecting drug resistance can be categorized into two groups: physical and biological components (Figure 2, Table 2). Physical components that interfere with the delivery and efficacy of CDDP include high cell density, fluidic shear stress, and the extracellular matrix (ECM). The biological component group consists of biochemical consequences of tumor growth (e.g., hypoxia and acidity) and noncancerous cells (e.g., stromal cells, tumor-associated fibroblasts, and immune cells). We summarize key experimental evidence of these factors in the following.

### 4.1. Physical Factors

The first obstacle of the TME that inhibits chemotherapy drugs from reaching tumor cells is the physical barrier that is composed of closely packed tumor cells. Experimental results from several studies using three-dimensional cell culture platforms have indicated the restricted diffusion capacity of various chemotherapy drugs, including CDDP into tumor tissues, resulting in reduced cytotoxicity [62,63]. Additionally, the interaction between rapid tumor growth, the surrounding ECM, and disorganized surrounding vessels can result in increased interstitial fluid pressure, also called fluidic shear stress [64]. An experimental study with a well-designed microfluidic platform demonstrated that increased shear stress can induce not only cancer stemness progression but also CDDP resistance in ovarian cancer cells [64]. Evidence based on this experimental model mimicking tumor behavior in the peritoneum indicated that shear stress might promote CDDP resistance through the activation of phosphatidylinositol 3-kinase/Akt (PI3K/Akt) signaling and ABC drug transporters in tumor cells.

The ECM, primarily composed of collagen, laminin, and fibronectin, is the major noncellular component of the TME [81]. The physical role of the ECM is to act as a scaffolding to maintain tissue structure and function. Accordingly, the ECM is a key player in tumor progression and chemotherapy drug resistance. Alterations in ECM stiffness and elasticity can establish a physical barrier hindering drug delivery to cancer cells. Additionally, studies have reported that interactions of the ECM with surrounding cells can promote chemotherapy drug resistance through the activation of survival proteins [6,7,65,82]. This so-called cell adhesion–mediated drug resistance (CAM-DR) is a type of chemoresistance mainly provoked by receptor-ligand interactions of the tumor cell with the TME. When tumor cells come into contact with the ECM or stromal cells, integrin-mediating signaling pathways can produce several antiapoptotic molecules and promote chemotherapy drug resistance. In a recent study with translational medicine, Senthebane and colleagues reported that the use of collagen- and fibronectin-deficient ECMs can synergistically increase cancer cell sensitivity to CDDP by 30%–50% [65]. The authors also suggested that the activation of PI3K/Akt signaling pathways induced by the ECM correlates to CDDP resistance in esophageal cancer cells.

### 4.2. Biological Factors

#### 4.2.1. Reduced Blood Flow

Compact aggregation of tumor cells and reduced blood flow can result in tumor tissue hypoxia. Low oxygen levels at the tumor site subsequently facilitate cancer cell stemness and multidrug transporter expression, thus resulting in CDDP resistance [66,67,68,69,70]. Preclinical studies have indicated that antiangiogenic drugs, such as bevacizumab, can improve the flow of tumor vessels (vascular normalization) and the delivery function of coadministrated chemotherapy drugs [83]. When these antiangiogenic drugs are used at lower doses, they can partially decrease blood vessel numbers in tumors, but enhance the function of tumor vessels with a more intact basement membrane [83]. More importantly, pivotal clinical trials have shown that patients with lung cancer receiving a bevacizumab plus CDDP-based regimen have longer survivals compared with those receiving chemotherapy alone (Table 3) [84,85]. These promising results substantially encourage the initiative to overcome CDDP resistance through the regulation of tumor vessel function. In addition to limited oxygen delivery, deficient nutrition supply from disorganized tumor vessels impels tumor cells into glycolysis and more acidic waste production [71,72]. This acidic TME can promote multidrug transporter expression and also reduce intracellular CDDP accumulation.

#### 4.2.2. Cellular Crosstalk within The Microenvironment

Intercellular interactions in the TME are also major factors that confer CDDP resistance on tumor cells through various mechanisms [86]. In addition to CAM-DR, interactions between stromal and tumor cells can also modify the ECM and secret growth factors, support tumor angiogenesis, suppress anticancer immune response, and therefore produce a TME niche that promotes drug resistance. Carcinoma-associated fibroblasts (CAFs) are the predominant cell type within the TME, and their associations with CDDP resistance have been widely explored [73,74,75,76,77,78]. In most studies using CAF coculture methods, tumor cells obtained CDDP resistance through CAF-secreted chemokines or growth factors, such as interleukin (IL)-6, IL-8, IL-11, insulin-like growth factor 1, or transforming growth factor-β (TGF-β). Similar to CAM-DR, these paracrine interactions can effectively activate several vital biological responses correlating to drug resistance, such as antiapoptosis signaling pathways, cancer cell stemness maintenance, and accelerated epithelial–mesenchymal transition.

Tumor-associated macrophages (TAMs) are also associated with tumor progression and chemotherapy drug resistance [87]. Two distinct phenotypes of macrophages exist, depending on the adjacent environments and tumor stages. M1-type macrophages can inhibit tumor growth through the induction of an inflammatory response. By contrast, M2-type macrophages support tumor progression by suppressing immune response. Phenotypes and functions of TAMs surrounding human cancer are similar to those of M2-type macrophages. Similar to CAFs, TAMs also promote tumor angiogenesis, enhance cancer cell stemness, remodel ECMs, and suppress host immune response. Numerous studies have shown that tumor CDDP resistance can be provoked by several TAM-secreted cytokines, such as IL-6 and type I interferon (IFN) [79,88]. Because of the active role of TAMs in regulating tumor progression, suppressing tumor CDDP resistance by therapeutically targeting TAMs might be feasible [79,80]. In tumor cells, colony stimulating factor 1 receptor (CSF-1R) signaling can mediate the polarization status of TAMs. In a recent study using a transgenic mouse model with breast cancer cells, Salvagno and colleagues showed that CSF-1R blockade is an effective way to enhance CDDP-induced anticancer activity, which is stimulated by an intratumoral type I IFN response [79]. These results indicate that regulating polarization states of TAMs can be a rational approach to resolve CDDP resistance in tumor cells.

It has become clear that secreted extracellular vesicles (EVs) can be responsible for intercellular communication in the TME. Increasing evidence has also shown that these secreted EVs within the TME are involved in the development of CDDP resistance in tumor cells [89,90,91,92]. EVs are a heterogeneous group of cell-derived vesicles, including exosomes with sizes ranging from 30 to 150 nm in diameter, and microvesicles from 150 to 1000 nm. The lipid bilayer of EVs encloses their biological contents, such as proteins, lipids, and genetic materials. In addition to direct exporting chemotherapy drugs, EVs can be able to regulate several biological processes, including drug resistance by transferring their containing biological materials to the target cells. Furthermore, Samuel and colleagues demonstrated that secreted EVs from ovarian cancer cells during CDDP treatment can lead to invasiveness and drug resistance in bystander cells [90]. These results suggest that stressed tumor cells with CDDP treatment can mediate CDDP resistance in other treatment naïve tumor cells through the communications with these vesicles. Beyond tumor cells, recent experimental results have revealed that secreted exosomes from CAFs can regulate CDDP resistance in tumor cells. Qix and colleagues showed that miR-196a wrapping in CAF-derived exosomes can enhance CDDP resistance in head and neck cancer cells [92]. Taken together, these results reveal that the intercellular crosstalk through secreted EVs in the TME might be a potential therapeutic target for overcoming CDDP resistance.

#### 4.2.3. Immune System

Cytotoxic CD8^+^ lymphocytes (CTLs) can secrete cytotoxic enzymes, such as perforine or granzyme, to induce tumor cell death [93,94]. However, tumor cells can escape CTL attacks if they activate immune checkpoint signaling pathways (e.g., cytotoxic T lymphocyte antigen-4 and programmed death-1). Recently, immune checkpoint inhibitors (ICIs) have exhibited their immune-mediated activity in eliminating tumor cells through interrupting coinhibitory signaling pathways in CTLs [95]. With the successful development of ICIs in clinical use, CTL activity against tumor growth has become an appealing research topic. Several studies have indicated the remarkable correlation between the presence of CTLs and CDDP resistance. In some studies analyzing tumor tissues, a high proportion of CTLs surrounding a tumor was significantly correlated with a superior response to chemotherapies [96,97]. Additionally, some preclinical studies have delineated the intricate connection between CTL-mediated tumor death and CDDP resistance. Wang and colleagues observed that intimate interactions between CTLs and CAFs are associated with CDDP resistance in ovarian cancer cells. Results from this study suggested that CTLs can abrogate CAF-mediated GSH metabolism and platinum resistance through the immunogenic action of IFN-β [98]. Moreover, the DNA damage property of CDDP can also stimulate costimulatory signaling pathways for CTL-dependent tumor death. In a study examining immunogenic effects of CDDP, Beyranvand and colleagues demonstrated that CDDP can enhance tumor cell death with the assistance of CTLs activated by CD80/86-mediated costimulation [99]. Recent well-designed clinical trials in patients with lung cancer have determined the clinical value of targeting CTLs in CCDP resistance (Table 3). Combination treatment with ICIs and platinum-based chemotherapies is associated with better survival outcomes than chemotherapy alone [100,101]. These promising results have encouraged a number of clinical trials in progress to evaluate the approach of combining therapeutic targeting of CTLs and CDDP-based therapies in patients with various types of cancer.

## 5. Ongoing Approaches to Overcome CDDP Resistance

Because CDDP has become the mainstay of anticancer treatment over the past two decades, numerous efforts have been devoted to clarify the mechanisms that participate in CDDP resistance. Initial molecular studies have directly focused on internal characteristics, such as drug transporters, detoxification enzymes, DNA damage repair systems, and apoptotic signaling pathways, of tumor cells. With recent developments in experimental methods, such as 3D culture platforms and syngeneic mouse models [7], studies investigating the interaction between tumor cells and their surrounding environments have emphasized the role of TME in tumor CDDP resistance. These observations yield a clearer picture of the tumor, which is a complex mixture composed of tumor and stromal cells and the ECM. More novel and useful therapeutic strategies for resolving CDPP resistance are expected to be invented because of this clear progress in tumor biology. In the TME, most stromal components can be manipulated through engineering to act against tumor growth because of their susceptible behaviors. Tumor-adjacent cells are unlikely to acquire resistance to therapeutic agents because of their stable genetic background. The unique compositions of the TME, which are quite different from the environments adjacent to normal tissue, also make the tumor vulnerable to TME-targeting therapies. These advantages all can be used to develop potential therapeutic strategies for overcoming tumor CDDP resistance. Recently, clinical benefits from combination treatments using platinum-based therapies and TME-targeting agents, such as antiangiogenic drugs and ICIs, have proven the considerable value of these agents for circumventing CDDP resistance in patients with cancer [84,85,101,102]. More potent combination treatments using novel TME-targeting agents and CDDP-based therapies for patients with various types of cancer should be explored.

The TME can impair CDDP efficacy in tumor cells through the following phenomena: limited drug delivery, cell death inhibition, drug inactivation, cell stemness promotion, or any combination of these factors. Combination therapies targeting different levels of resistance mechanisms consequently appear to be a reasonable way to combat CDDP resistance in tumor cells. Several novel agents targeting these TME factors, including CXC chemokine receptors, focal adhesion kinase, and the signaling pathway of fibroblast growth factor, TGF-β, immune checkpoints, and CSF1R have been developed in the clinical trial stage. Among these factors, the activation of CSF-1R signaling in macrophages can lead to the M2 polarization status of TAMs, which might impart the TME with an immunosuppressive characteristic. Although clinical trials with CSF1/CSF1R antagonists are in early phases currently, a recent study highlighted the predictive value of *CSF1R* genotypes in this type of TME-targeting therapy. In this study, we found that the *CSF1R* c.1085A>G genetic variant, which is present in approximately 40% of the East Asian population, can regulate the polarization status and function of macrophages [102]. Macrophages with this *CSF1R* genetic variant are resistant to CSF-1 stimulation but sensitive to CSF-1R inhibitors. These results suggest that the *CSF1R* c.1085A>G genetic variant might be a potential biomarker that can be used in targeting CSF-1R signaling for cancer treatments. Reliable biomarkers of stromal activity, immunogenic properties, and cancer cell stemness features are also essential to support successful therapeutic strategies targeting the TME in most types of cancer treatments.

In addition to the TME, some promising experimental therapies targeting intrinsic resistance mechanisms in tumor cells have been reported. As mentioned above, the Cu transporters are important regulators of CDDP resistance, and several studies have established the interventions of these transporters, especially CTR1. Because the transportation of platinum-based drugs closely interacts with Cu homeostasis regulation, early clinical trials are underway to evaluate the effectiveness of the Cu chelators, trientine, and tetrathiomolybdate, in enhancing CTR1 expression levels and reducing CDDP resistance in some cancer types. Although clinical responses in the first study to evaluate the efficacy of trientine in platinum-refractory tumors were unsatisfactory, the mechanistic basis derived from this study suggests the potential utilization of these chelators [103]. In this early clinical trial, patients who had low Cu or ceruloplasmin levels during trientine treatment exhibited better clinical outcomes. Accordingly, investigations into targeting these transporters to enhance CDDP efficacy should be undertaken.

The advancement of nanotechnology offers a possible method for the management of CDDP resistance. Advantages of anticancer drugs with a nanocarrier-based delivery system include selective targeting to tumor cells, reduced systemic toxicity, enhanced cellular uptake, and more drug accumulation inside the tumor cells [104,105]. In general, nanoparticles enter cells through endocytotic mechanisms, which are constantly maintained in CDDP-resistant tumor cells. Accordingly, nanoparticles can effectively deliver platinum into CDDP-resistant tumor cells and increase cytotoxicity. Additionally, dimensions of nanoparticles vary in the range of 50–200 nm, which causes the easy accumulation of these carriers in tumor sites because of enhanced permeability and retention effects. This tendency of drug accumulation toward tumor cells can thereby increase anticancer efficacy and minimize systemic toxicity. Several types of nanoparticles have been developed to deliver platinum-based drugs, namely carbon nanotubes, gold nanoparticles, liposomes, and polymeric micelles [106]. With promising results from preclinical studies [106,107,108,109], the clinical benefits of nanomaterial-based CDDP formulations, such as Nanoplatin^TM^, Aroplatin^TM^, Lipoplatin^TM^, and SPI-077, are being investigated with clinical trials currently. Moreover, nanotechnology-based drug delivery systems can provide the codelivery of CDDP with other anticancer drugs or agents targeting resistance mechanisms of tumor cells [110]. Therefore, these carriers can simultaneously release multiple anticancer agents that have synergistic cytotoxic effects on tumor cells and assist in overcoming CDDP resistance. Special attention should also be devoted to the development of medical materials or complexes that facilitate the delivery of chemotherapy drugs and enhance their cytotoxic effects.

## 6. Future Perspectives and Conclusions

In summary, CDDP has demonstrated major clinical success in patients with various types of cancer for more than two decades; however, drug resistance of tumor cells has remained the main impediment for the clinical use of CDDP. Preclinical and clinical studies have showed that CDDP resistance is a complex biological phenomenon mediated by inner adaptive mechanisms of tumor cells responsive to not only CDDP stimulation but also the interaction from any TME component. Accordingly, developing technologies that fully mimic the intricate microenvironment of tumor cells and providing an approach for designing practical therapies for CDDP resistance are of paramount importance. Because drug resistance of tumor cells is always the result of multiple affecting factors, combination therapies simultaneously targeting multiple mechanisms that lead to CDDP resistance should be preferentially explored. However, when searching for potent therapeutic targets, one should consider that the identification of ideal biomarkers is also equally critical. Practical biomarkers can be useful in identifying feasible populations, predicting treatment outcomes, and monitoring disease responses for therapeutic target-directing therapies. Additionally, the development of biomaterials, which facilitates the delivery of chemotherapy drugs or the directly therapeutic targeting of resistance mechanisms, can be of great assistance in overcoming CDDP resistance. For example, several potent nanoparticle-based CDDP formulations in clinical development are in the stage of clinical trial. Determining how to intimately incorporate emerging knowledge from tumor biology, molecular pharmacology, and material engineering is a key step in the approach to overcoming CDDP resistance.

## Figures and Tables

**Table 1:** Intracellular regulators of cisplatin resistance with clinical implication.

Regulator	Action Mechanism	Relevance to CDDP Resistance	Reference
Cellular uptake			
CTR1	Membrane copper transporter	1. Low expression levels in CDDP-resistant cancer cells. 2. Correlation between CTR1 expression levels and intracellular platinum concentration. 3. Copper chelators enhance CDDP efficacy in vitro and in vivo. 4. Low expression levels in tumors predict poor clinical efficacy of CDDP.	[16,17,18,19,20,21]
CTR2	Membrane copper transporter	1. The induction of CTR1 cleavage. 2. High expression levels in tumors predict poor clinical efficacy of CDDP.	[22,23,24]
OCT2	Organic cation transporter	Low expression levels in tumors predict poor clinical efficacy of CDDP.	[25]
Cellular export			
ATP7A/ATP7B	Copper-exporting P-type ATPase	1. High expression levels in CDDP-resistant cancer cells. 2. High expression levels in tumors predict poor clinical efficacy of CDDP.	[21,26,27,28,29]
MRP2	ATP-binding cassette multidrug transporter	1. High expression levels in CDDP-resistant cancer cells. 2. High expression levels in tumors predict poor clinical efficacy of CDDP.	[30,31,32]
Drug inactivation			
GSH	Intracellular electrophiles scavenger	1. High expression levels in CDDP-resistant cancer cells. 2. High expression levels in tumors predict poor clinical efficacy of CDDP.	[33,34,35]
Metallothionein	Detoxification enzyme of a heavy metal	1. High expression levels in CDDP-resistant cancer cells. 2. High expression levels in tumors predict poor clinical efficacy of CDDP.	[36,37]
DNA damage repair			
ERCC1	NER	1. High expression levels in CDDP-resistant cancer cells. 2. High expression levels in tumors predict poor clinical efficacy of CDDP.	[38,39,40,41,42,43,44,45]
XPF	NER	1. High expression levels in CDDP-resistant cancer cells. 2. High expression levels in tumors predict poor clinical efficacy of CDDP.	[46]
BRCA1/BRCA2	HR	*BRCA1/2*-mutated tumors correlate to good responders to CDDP.	[47,48]

CDDP: cisplatin, CTR1: copper transporter 1, CTR2: copper transporter 2, OCT2: organic cation transporter 2, ATP7A: copper-transporting ATPase 1, ATP7B: copper-transporting ATPase 2, ERCC1: DNA excision repair protein ERCC-1, XPF: DNA repair endonuclease XPF, BRCA1: breast cancer type 1 susceptibility protein, BRCA2: breast cancer type 2 susceptibility protein, MRP: multidrug resistance-associated protein, GSH: glutathione, NER: nucleotide excision repair, HR: homologous recombination.

**Table 2:** Tumor microenvironment factors reported with cisplatin resistance.

Factor	Action mechanism	Experimental result	Reference
Physical			
Physical barriers	Limit penetration of CDDP into tumors	Decreased CDDP accumulation in tumor cells	[62,63]
Fluidic shear stress	Activation of PI3K/Akt signaling and ABC drug transporters	Cancer stemness progression and CDDP resistance induced by fluidic shear stress	[64]
ECM	1. Limited CDDP diffusion 2. The activation of survival signals through the interaction with tumor cells	Increased cancer cell sensitivity to CDDP in collagen- and fibronectin-deficient ECMs	[65]
Biological			
Hypoxia	Increased cancer cell stemness and multidrug transporter expression	Increased CDDP resistance in low oxygen levels	[66,67,68,69,70]
Acidity	Increased multidrug transporter expression	Increased CDDP resistance in acidic conditions	[71,72]
CAF	1. CAF-secreted growth factors or cytokines affecting cell apoptosis or intrinsic drug resistance 2. Metabolism of CAFs regulated by effector T-cells	1. Increased CDDP resistance by CAF-secreted cytokines such as IL-6, IL-8, IL-11, insulin-like growth factor 1, and TGF-β 2. CAFs-mediated GSH metabolism and platinum resistance abrogated by cytotoxic T cells	[73,74,75,76,77,78]
TAM	Secretion of cytokines by TAM in an M2 polarization state	Increased CDDP resistance by TAM-secreted cytokines such as IL-6 and type I interferon	[79,80]

CDDP: cisplatin, PI3K: phosphatidylinositol 3-kinase, ABC: ATP-binding cassette transporter, ECM: extracellular matrix, CAF: carcinoma-associated fibroblast, TAM: tumor-associated macrophage, IL: interleukin, TGF: transforming growth factor, GSH: glutathione.

**Table 3:** Novel agents targeting microenvironment components that achieve clinical benefits in combination with a cisplatin-containing regimen.

Drug	Category	Major target	Clinical benefit	Reference
Bevacizumab	Angiogenesis antagonist	Vascular endothelial growth factor A	1. In the AVAil study, combination therapy (cisplatin, gemcitabine plus bevacizumab) prolonged PFS (HR = 0.82; *p* = 0.03) in first-line therapy for patients with advanced nonsquamous nonsmall-cell lung cancer compared with the control group (cisplatin plus gemcitabine). 2. In the AVAPERL study, combination therapy (cisplatin, pemetrexed plus bevacizumab) prolonged PFS (HR = 0.48; *p* < 0.001) in first-line therapy for patients with advanced nonsquamous nonsmall-cell lung cancer compared with the control group (cisplatin plus pemetrexed).	[84,85]
Pembrolizumab	Immune check point inhibitor	Programmed cell death protein 1	In the KEYNOTE-189 study, combination therapy (cisplatin, pemetrexed plus pembrolizumab) increased OS at 12 months (HR = 0.49; *p* < 0.001) in first-line therapy for patients with advanced nonsmall-cell lung cancer compared with the control group (cisplatin plus pemetrexed).	[101]

PRS: progression-free survival, HR: hazard ratio, OS: overall survival.
